# Integrating Psychosocial Care into Orthopedic Settings: A Qualitative Study of Provider Perspectives

**DOI:** 10.5334/ijic.7579

**Published:** 2023-12-06

**Authors:** Mira Reichman, Ellie A. Briskin, Brooke A. Duarte, Ana-Maria Vranceanu, Victoria A. Grunberg

**Affiliations:** 1 University of Washington, Department of Psychology, Seattle, WA, USA; 2 Center for Health Outcomes and Interdisciplinary Research, Department of Psychiatry, Massachusetts General Hospital, Boston, Massachusetts, USA; 3 Suffolk University, Department of Psychology, Boston, Massachusetts, USA; 4 Harvard Medical School, Boston, Massachusetts, USA; 5 Division of Newborn Medicine, MassGeneral for Children, Boston, Massachusetts, USA

**Keywords:** Orthopedic trauma, integrated care, psychosocial services, patient satisfaction

## Abstract

**Introduction:**

Approximately 50% of persons with orthopedic injuries experience psychosocial distress (e.g., depression, anxiety), which can predict chronic pain and disability. Offering psychosocial services in orthopedic settings can promote patient recovery. This study explores health care professionals’ perceptions of and recommendations regarding integrated psychosocial care for orthopedic settings.

**Methods:**

We conducted 18 semi-structured focus groups with 79 orthopedic health care professionals (e.g., surgeons, residents, nurses) across three Level I Trauma Centers. This secondary data analysis used the evidence-based Rainbow Model of Integrated Care framework to structure hybrid inductive-deductive qualitative data analysis.

**Results:**

Orthopedic health care professionals identified potential benefits to psychosocial service integration across all dimensions of integration (i.e., clinical, professional, organizational, system, functional, and normative). These benefits included increased patient satisfaction with care, decreased burden on medical providers to manage patient distress, and decreased healthcare utilization costs. They also identified barriers (e.g., fast-paced clinic flow, mental health stigma) and offered recommendations to address barriers across dimensions of integration.

**Conclusion:**

Integrated psychosocial care for orthopedic trauma patients has the potential to improve patient recovery and long-term physical and mental health outcomes. This work identifies strategies to inform the development and implementation of initiatives to integrate psychosocial services within orthopedic settings.

## Introduction

Orthopedic traumas are a leading cause of disability globally, resulting in chronic pain and functional limitations in up to 50% of patients [1,2]. Recovery trajectories after orthopedic injuries can contribute to fear of activity and depressed mood given that patients face a prolonged period of inactivity while their bones and soft tissues heal [3]. Psychological distress can interfere with re-building strength and mobility, thereby inhibiting patients’ recoveries and increasing risk for chronic pain and disability [4,5]. In fact, depression, anxiety, posttraumatic stress, and catastrophic thinking are among the strongest predictors of chronic pain and disability, irrespective of injury type and severity [6,7,8,9,10,11].

With 40–50% of orthopedic patients experiencing depression, anxiety, or posttraumatic stress 3 months to 2 years after an injury [12], psychosocial services are needed to improve patients’ well-being and physical recoveries [13,14]. However, orthopedic care remains largely biomedical and few patients have access to or participate in psychosocial care [10,15]. Orthopedic medical providers have described important barriers that may prevent them from referring patients to psychosocial services [16]. These include: 1) limited understanding or acknowledgement of psychosocial factors that impact recovery; 2) stigma associated with discussing mental health; 3) limited knowledge of available psychosocial services; and 4) lack of time within fast-paced clinic flows [16].

Integrated behavioral health care models provide an innovative solution to overcome these barriers. In line with the biopsychosocial model of healthcare [17,18], these collaborative models blend medical and psychosocial services [19]. Integrated care models have been successfully implemented across several medical settings, including primary care and specialized settings (e.g., rehabilitation, cardiology) [20,21,22]. They have shown to be effective in promoting patient health outcomes, increasing patient satisfaction with care, and decreasing healthcare costs [23,24]. Despite the value of integrated behavioral care models, they have yet to be implemented in orthopedic settings. A thorough understanding of individual, organizational, and systemic factors that would influence integrated care initiatives in orthopedic settings is an important first step to inform implementation.

The Rainbow Model for Integrated Care, a theoretical framework informed by literature review and expert consultation [25,26], delineates the multi-level dimensions that influence implementation of integrated care (see Table 1). The model describes six dimensions of integrated care – four of which (system, organizational, professional, and clinical) are positioned along levels where integration can take place, (macro, meso, and micro), and two of which span these levels (functional and normative). In a systematic scoping review of available integrated care models, the Rainbow Model was one of five theoretical models recommended for coordination of care with external partners and the only model recommended for a specific disease or setting [27]. Therefore, the Rainbow Model is a well-suited, comprehensive, evidence-based framework to structure the exploration of factors that influence approaches to integrating psychosocial services into orthopedic trauma care. Very few studies have used theoretical frameworks to structure the examination of factors that impact integrated care in specialty medical settings [28,29,30], and none have done so within orthopedics.

**Table 1:** Six Dimensions of the Rainbow Model for Integrated Care (Valentijn et al., 2013).


DIMENSION	DEFINITION

System integration (Macro level)	Rules, policies, and structures that promote delivery of holistic healthcare putting individuals’ needs at the center.

Organizational integration (Meso level)	Inter-organizational relationships (e.g., contracting, strategic alliances, knowledge networks) to deliver comprehensive services to a defined population.

Professional integration (Meso level)	Inter-professional partnerships (between or within organizations) based on shared roles, responsibilities and accountability to deliver a comprehensive continuum of care to a defined population.

Clinical integration (Micro level)	Coordination and delivery of person-focused care within a single setting and a single process across disciplines.

Functional integration (Across levels)	Key support functions and activities (e.g., financial, management and information systems) to coordinate and support accountability and decision-making between organizations and professionals.

Normative integration (Across levels)	Development and maintenance of common mission, vision, values and culture between professionals, groups, and organizations.


Integrating psychosocial care into orthopedic settings requires multidisciplinary support, and the buy-in of orthopedic medical care providers (e.g., surgeons, residents, nurses). Across other medical settings, ideological differences associated with specialization, financial and billing issues, and supportive leadership have been identified as barriers to integrated care [20]. However, the relevance of these multi-level factors for integrated care in orthopedics remains largely unknown. Orthopedic providers are uniquely positioned to offer insight into the relevant clinical, professional, organizational, and system factors that could promote or hinder integrated care efforts. A qualitative approach, informed by the Rainbow Model, is well-poised to generate an in-depth understanding of orthopedic provider perspectives on the multiple, interconnected dimensions that influence the implementation of integrated care in orthopedic settings [31,32].

The purpose of this secondary data analysis is to examine orthopedic health care professionals’ perspectives regarding integrated psychosocial services within orthopedic trauma care. Findings will help to inform how clinical, professional, organizational, system, functional, and normative factors can be leveraged to inform the implementation of integrated psychosocial care in orthopedic settings.

## Methods

We conducted this study across three geographically diverse Level I Trauma Centers within academic medical centers in the United States (Austin, TX (Site A); Lexington, KY (Site B); and Boston, MA (Site C)). We conducted 18 semi-structured qualitative focus groups with exit interviews over live video with *N* = 79 orthopedic health care professionals to understand their perceptions of integrating psychosocial services into orthopedic settings. Site C Institutional Review Board approved all study procedures.

### Participants

We recruited participants across sites A, B, and C between October and November of 2020. In collaboration with “surgeon champions” (i.e., study ambassadors), we presented the study to the three orthopedic departments. We recruited orthopedic health care professionals across disciplines (e.g., surgeons, nurses, physical therapists) to capture a diversity of perspectives. After each recruitment presentation, we distributed a screening survey electronically through Research Electronic Data Capture [33] to potential participants to determine eligibility and provide detailed information about the study. Completion of this screening survey constituted implied consent to participate in the study.

We distributed the screening survey to 94 orthopedic health care professionals, 88 of whom (94%) completed the survey. Seventy-nine of those (90%) participated in qualitative data collection. Nine participants who consented (10%) did not participate in focus groups due to scheduling conflicts. The final sample of participants included 20 attending surgeons; 28 residents; 10 (total) nurse practitioners, registered nurses, and physician assistants; 13 medical assistants; five (total) physical therapists and social workers; and three research fellows (Table 2). Surgeons and residents were mostly men (92%), and other healthcare professionals were mostly women (68%). Participants were predominantly white (73% of surgeons and residents; 71% of other healthcare professionals). Half of the participants across roles reported mental health training during their medical training or continuing education (50% of surgeons and residents; 52% of other healthcare professionals).

**Table 2:** Participant Characteristics.


VARIABLE	SURGEONS AND RESIDENTS (*N* = 48)	NON-PHYSICIAN HEALTHCARE PROFESSIONALS (*N* = 31)

**Gender**		

Men	92% (44)	32% (10)

Women	6% (3)	68% (21)

Other	2.1% (1)	0% (0)

**Age in years**		

25–39	67% (32)	65% (20)

40–55	27% (13)	32% (10)

56–75	6.3% (3)	3.2% (1)

**Race**		

White	73% (35)	71% (22)

Black	8% (4)	10% (3)

Asian	13% (6)	0% (0)

Multiracial or other	6% (3)	19% (6)

**Ethnicity**		

Hispanic or Latino	2% (1)	39% (12)

Non-Hispanic or Latino	98% (47)	61% (19)

**Household income in USD**		

20,001–50,000	0% (0)	29% (9)

50,001–100,000	44% (21)	23% (7)

100,001–200,000	17% (8)	39% (12)

200,001–300,000	4% (2)	6% (2)

300,001–400,000	0% (0)	0% (0)

400,001–500,000	4% (2)	0% (0)

500,001–750,000	25% (12)	3% (1)

>750,000	6% (3)	0% (0)

**Self-reported mental health training**		

Yes	50% (24)	52% (16)

No	50% (24)	48% (15)


### Qualitative Data Collection

We conducted 18 focus groups with individual exit interviews (*N* = 76 participants) over secure live videoconferencing (i.e., Zoom). We assigned participants to focus groups based on their professional role (i.e., surgeon groups, resident groups, nurse groups, etc.). Each group included 4–8 participants to promote engagement and dialogue. In some cases, we combined participants of several roles (e.g., nurse practitioners and physician assistants) to reach the target number of participants per group. Focus group discussions lasted 60 minutes and were followed by 10-minute individual exit interviews using the “break-out room” function in Zoom. We interviewed department chiefs (*N* = 3) individually in semi-structured 30-minute interviews given that their presence in focus groups could influence the opinions expressed by others.

Our multidisciplinary team of psychologists, orthopedic medical care providers, and an implementation science expert developed the semi-structured focus group script. The focus group script (Table 3) covered topics including participants’ perceptions of the psychosocial needs of orthopedic patients and factors that might promote or hinder integrated care in orthopedic settings. The open-ended exit interviews provided participants with the opportunity to express any additional perspectives that they did not express in the group setting. Predoctoral and postdoctoral research fellows in psychology with training from our multidisciplinary team conducted the focus groups, exit interviews, and department chief interviews. We audio recorded and transcribed verbatim all focus groups, exit interviews, and department chief interviews.

**Table 3:** Semi-structured focus group script domains and questions.


DOMAINS	QUESTIONS

Perceptions of the psychosocial needs of orthopedic patients	What comes to mind when you think of the terms “psychological, mental health, or behavioral concerns”? How often do you notice psychological, mental health, or behavioral problems in your patients? Do you formally assess or screen patients for psychological problems? What do you think about the role of these factors in the recovery trajectory of your patients?

Comfort addressing psychosocial factors in patients with orthopedic trauma	How do you address mental or behavioral health problems that you notice in your patients? Do you ever refer or initiate the connection of patients to mental or behavioral health services? What mental and behavioral health resources are you aware of that are potentially available to your patients? What would be an ideal scenario for addressing mental health factors for your patients?

Perspectives on integration of psychosocial care integration in orthopedic departments	How supportive are you of integrating psychosocial care within the orthopedic practice? What do you see as the most significant barriers to the integration of psychosocial care within orthopedic departments?

Individual exit interview (optional)	Is there anything that you would like to share that is relevant to the discussion from the focus group that you did not share in the focus group for any reason? How was your experience in the focus group today?


### Qualitative Data Analysis

We used the Rainbow Model of Integrated Care framework to structure a hybrid inductive-deductive qualitative secondary data analysis [34] and explore orthopedic health care professionals’ perspectives on integrating psychosocial care in orthopedic settings. The six dimensions of integrated care from the Rainbow Model served as a priori defined codes. In a deductive approach, two independent coders read all transcripts and organized the qualitative data relevant to our research question within these six codes using NVivo software [35]. Discrepancies in coding between the two coders were resolved by discussions with the broader research team to reach consensus.

After coding, the research team took a collaborative and inductive approach to data interpretation. We examined the data coded within each dimension and summarized insights that characterize participants’ perspectives. We identified themes that describe participants’: 1) perceptions of different approaches to integrating psychosocial services in orthopedic care, and 2) recommendations to maximize success of integration initiatives.

## Results

Tables 4 and 5 display the themes that characterize orthopedic health care professionals’ perceptions of integrated psychosocial services and recommendations for integrated care initiatives within each dimension of the Rainbow Model. Below, we describe several key themes and illustrative quotations.

**Table 4:** Orthopedic health care professionals’ positive and negative perceptions by integrated care dimension.


DOMAIN	THEMES

Clinical integration (micro level):	Positive perceptions: Addressing psychosocial factors would increase patient satisfaction with care Integrated care would promote accessibility to mental health services for patients Negative perceptions: Uncertainty whether patients would accept psychosocial services in traditionally biomedical setting Uncertainty whether patients have interest in psychosocial services given stigma Offering psychosocial services at clinic would be time-consuming for patient Integrated care would demand time and resources from medical providers Offering psychosocial services might instigate or exacerbate patient anxiety

Professional integration (meso level):	Positive perceptions: Value would be generated for providers having service to offer patients in distress Psychosocial services may reduce burden on providers by minimizing post-op calls Interest in learning about psychosocial aspects of orthopedic traumas

Organizational integration (meso level):	Positive perceptions: Offering psychosocial services would fill a critical gap in patient care Psychosocial service integration could promote organizational efficiency (e.g., less follow-up visits, reduced provider burn-out) Negative perceptions: Changes made to clinic procedures need to be approved by hospital

System integration (macro level):	Positive perceptions: Psychosocial service integration could help reduce healthcare system burden (i.e., reduce healthcare costs, improve healthcare efficiency)

Functional integration (micro, meso, and macro levels):	Negativeperceptions: More providers would further fragment care and disrupt communication Lack of funding for psychosocial services/providers

Normative integration (micro, meso, and macro levels):	Positive perceptions: Existing collaborative culture to bolster multidisciplinary team cohesion Value placed in continued learning, research, and professional growth Shared mission across multidisciplinary providers (i.e., wanting patients to get back to their activities) Distress is common in orthopedic settings and that distress shapes patient recovery and experience of pain Addressing psychosocial factors is in line with valued patient-centered, holistic care Alignment of psychosocial services with medical treatment goals and recommendations (e.g., engage in physical therapy despite pain) Negative perceptions: Psychosocial factors are not relevant to orthopedic care and lack of interest in addressing them Misconceptions about mental health (e.g., belief it is patient’s choice to have a positive mindset or not) Mental health cannot be improved through intervention Discomfort discussing mental health among orthopedic medical providers


**Table 5:** Orthopedic health care professionals’ recommendations by integrated care dimension.


DOMAIN	THEMES

Clinical integration (micro level):	Assess psychosocial factors during patients’ earliest visits Dedicate sufficient time in clinic flow for psychosocial service delivery Protect patient privacy when receiving psychosocial services Ensure that integrated care does not cause significant delays to providers Make psychosocial services available to all patients regardless of risk to help normalize distress Tailor psychosocial services to patient needs (e.g., brief versus long-term) Tailor services to patient socio-economic status and health literacy needs (e.g., remote option, Zoom tutorial) Utilize frequent communication and reminders to support patient engagement (e.g., texts, emails, check-ins)

Professional integration (meso level):	Secure leadership and influential stakeholder support for integration to facilitate provider buy-in Equip medical providers with psychosocial knowledge to enhance inter-professional communication Equip psychosocial service providers with orthopedic knowledge to enhance inter-professional communication Focus on common provider treatment goals (e.g., patient wellbeing and recovery) to promote teamwork Ensure that roles and responsibilities of multidisciplinary healthcare teams are clear Have medical providers perform warm hand-offs to psychosocial service providers

Organizational integration (meso level):	Develop safety protocols for patients who require higher levels of psychiatric care

System integration (macro level):	Enhance inclusivity and accessibility of psychosocial services by addressing barriers to care engagement (e.g., housing, food security, transportation, internet, health insurance)

Functional integration (micro, meso, and macro levels):	Ensure ongoing communication among collaborating providers regarding patient outcomes and integration progress Have designated staff to help coordinate multidisciplinary services Use existing clinic information and record-keeping systems (e.g., OR boards, EMR) Streamline psychosocial screening and referral procedures (e.g., order in EMR) Use electronic resources for education and communication with providers Ensure that providers and/or interpretative services are available for patients who speak different languages Ensure that referrals to outpatient psychosocial services are accessible for patients Have office space designated for psychosocial services

Normative integration (micro, meso, and macro levels):	Educate orthopedic providers on the psychosocial aspects of pain and injury to support a shared vision Provide empirical evidence that integrated care can improve patient outcomes Ensure services are culturally sensitive, inclusive, and individualized to patient-specific needs Establish shared communication styles among team members (e.g., concise, data-driven)


### Clinical Integration

Participants expressed that making psychosocial services a seamless component of post-injury care would support patients’ wellbeing by increasing their access to mental health care. They noted that this may increase patient satisfaction with clinic care. Participants also expressed some concerns to integrated care. They explained that mental health can be stigmatizing and therefore were unsure whether patients would be open to psychosocial care in a traditionally biomedical setting. As one medical assistant expressed, *“It’s such a taboo issue some people feel, and some people have said ‘Well I’m not,’ you know, ‘I’m here for my shoulder pain, I’m not here to talk about my feelings’.”* To address these concerns, participants recommended that clinic teams ensure patient privacy during any in-house psychosocial service delivery. Participants recommended that psychosocial services be offered to all patients (regardless of perceived need) to normalize psychosocial challenges and decrease stigma. Participants explained that to successfully integrate psychosocial care into these fast-paced settings, it is important that services do not interfere with or cause delays to the standard clinic flow.

### Professional Integration

Participants expressed interest in learning from multidisciplinary professionals about the intersections between psychosocial factors and orthopedic trauma. They shared that providing psychosocial care could help reduce post-injury admissions and contacts, therefore benefitting providers as well as patients. As one nurse practitioner explained, “*Decreasing post-op surgery calls would be a huge sell… it affects all of us, right, and so you could sell it that way, and again, that goes back to the patients having a better recovery and successful outcome… And this will help decrease phone calls post-op maybe, awesome*.” Participants recommended that orthopedic providers and psychosocial providers gain knowledge of each other’s disciplines to promote interprofessional collaboration and patient wellbeing. As one resident said, *“[Mental health providers] have to, you know, tease out the differences [in post-injury movement restrictions], and all of them are specific motion restrictions that, you know, even physical therapists can have challenges with*.” Participants also noted that integrated care initiatives would be most effective if supported by surgical leadership and other influential stakeholders. They suggested that orthopedic medical care providers engage in warm hand-offs when introducing patients to psychosocial care providers to promote patient uptake. As a nurse described, “*If it came from their own surgeon or something like that… a lot of them think like ‘That’s God,’ like, they’re up on that pedestal so, if they, you know, they’re recommending it, then ‘sure*.’”

### Organizational Integration

Participants believed that integrating psychosocial care into orthopedic trauma settings would fill a critical gap and could increase hospital efficiency and outcomes, allowing resources to be allocated more appropriately and effectively. As one social worker shared, “*From a needs-perspective, we’re talking about behavioral needs, health needs being beyond what we have the capacity to do [at this hospital], like, we need anything that’s being done that is potentially facilitating more patients with behavioral health needs—having those needs met.”* On the other hand, participants expressed some hesitancy regarding the feasibility of enacting organizational changes given hospital regulations and lengthy approval processes. Participants recommended including safety protocols into integrated care initiatives to connect patients who require a higher level of psychiatric care to appropriate resources.

### System Integration

Participants suggested that connecting orthopedic patients with psychosocial services during post-injury care could increase healthcare efficiency and decrease costs. As one resident explained, “*Coping—so less coming back to an urgent care, less coming back to the emergency room, less, you know, coming more to scheduled visits, and being able to, kind of, be on that timeline that you expect for the injury itself if they didn’t have a psychosocial thing going on, on top of that*.” Participants stated that systemic barriers may impact engagement in psychosocial care (e.g., housing and food security, transportation, internet, and health insurance). They recommended addressing these barriers to improve access to psychosocial services. One research personnel highlighted how accessible services would fill a needed gap: *“The orthopedic trauma patient population in general has been shown to, you know, have high prevalence of mental illness, substance abuse, they’re often low socioeconomic status that don’t have access to resources like this, so I think [integrated care] will hopefully do some good and have some impact where it’s desperately needed.”*

### Functional Integration

Participants highlighted that communicating with multiple health care professionals about patients’ needs and care may disrupt or complicate care. They also expressed that allocating funding for integrated psychosocial care may be difficult. As one surgeon noted, *“Like, who’s going to pay for the psychologist? … That’s what our boss is going to look at, you know, they’re going to look at the bottom—bottom dollar, you know.”* Participants recommended that psychosocial providers standardize screening and referral procedures and use existing clinic communication systems (i.e., electronic medical record, white board systems) to communicate with clinic staff. They also suggested that a staff member be designated to coordinate multidisciplinary services. As one resident shared, *“I think, personally, the concern for me would be, is this going to be an extra onus on the surgeon, the staff, or us as residents… [but] I don’t think it’s going to be an issue… if you have a well-integrated coordinator that helps, you know, get these people to the resources and, you know, referrals, and things like that.”*

### Normative Integration

Participants identified values that they believe can contribute to a common mission for multidisciplinary teams, such as continued learning and shared commitment to patient recovery and holistic wellbeing. Participants endorsed a variety of perceptions regarding the importance of addressing psychosocial factors in orthopedic settings. Some expressed the belief that mental distress does affect patient recovery. Others expressed discomfort discussing mental health, misconceptions about mental illness, and beliefs that psychosocial factors are not relevant to orthopedic care. One resident shared, *“I think it is kind of sad that [mental health] isn’t a topic that we really talk about… I think the more that we as orthopedic surgeons can help them break this stigma surrounding mental health and the more that our patients get that sense that we care about them, not just as a broken bone but as a person, I think that is a step in the right direction.”* Participants recommended that orthopedic health care professionals be educated on the psychosocial aspects of orthopedic pain and injury. They suggested using empirical evidence to demonstrate the value of integrated care initiatives to improve outcomes.

## Discussion

Psychosocial risk factors including depression, anxiety, posttraumatic stress, and catastrophic thinking are prevalent among individuals with acute orthopedic injuries and can contribute to chronic pain and disability [4,7,12]. Still, tailored psychosocial services remain largely unavailable for patients who experienced orthopedic injuries [10,15]. As evidenced in other specialty medical settings, integrated behavioral care can help address the unmet psychosocial needs of patients [19,20,21]. Informed by the Rainbow Model of Integrated Care [25,26], we conducted focus groups with orthopedic health care professionals to learn about their perceptions of and recommendations for integrating psychosocial care into orthopedic settings. Understanding their perspectives in-depth informs clinical initiatives to integrate psychosocial services into orthopedic settings.

Orthopedic health care professionals shared their perceptions of the potential value of integrated care and barriers that are important to consider across all levels, which align with research findings from other specialty medical settings. At the clinical level, participants acknowledged that psychosocial distress is prevalent and were open to integrated care to support the holistic wellbeing of their patients. They explained that limited time with patients, the fast-paced nature of these settings, and mental health stigma are key barriers to address—findings which are consistent with prior work [16]. At the professional level, participants endorsed limited understanding of the evidence that psychosocial factors impact pain and disability outcomes. They also noted that they would not feel comfortable or do not have the training to discuss mental health with patients. This finding is in line with prior literature indicating that ideological differences associated with specialization is a barrier to integrated care in specialty medical settings [20]. However, participants acknowledged that psychosocial services could improve patients’ recoveries, wellbeing, and satisfaction with care, as well as the effectiveness and efficiency of care. This perception is in line with valued patient-centered care (normative dimension) and suggests a point of intervention to promote buy-in from all health care professionals. Their belief that psychosocial services may help promote organizational effectiveness (organizational dimension) and healthcare efficiency, such as reduced readmissions or costs (systemic dimension), provide compelling support for large-scale investment of integrated care initiatives.

Orthopedic health care professionals shared recommendations to promote the feasibility, acceptability, and appropriateness of integrated care initiatives in orthopedic settings, all of which constitute novel contributions to the literature given their specificity to orthopedic settings. At the clinical level, it is important to assess psychosocial factors during patients’ earliest visits, protect patient privacy when participating in psychosocial services, and tailor psychosocial services to individual patient needs (e.g., health literacy). At the professional level, they noted that educational programs that provide information about psychosocial factors and care could help multidisciplinary collaboration. Prior work suggested that orthopedic health care professional prefer that educational materials are delivered concisely, electronically, and with strong evidence [36]. At the organizational level, participants in our study recommended having safety protocols in place for patients who require higher levels of psychiatric care. At the systemic level, they indicated a need to address structural barriers that interfere with treatment engagement (e.g., transportation, internet, health insurance). Across all levels, participants noted that a shared frame of reference and values (i.e., normative integration) and efficient and effective systems of coordination (i.e., functional integration) among professionals would promote integration. Taken together, the success of multi-level integration is dependent on communication, shared beliefs and values, resources, personnel, and leadership support.

Our study is the first to use the Rainbow Model to structure qualitative analyses to inform integrated care in a specialty medical setting. This framework allowed us to consider all dimensions and levels that influence integrated care as well as to align our study with previous literature exploring barriers and facilitators to integrated care across other settings. In addition, we recruited a large sample across three academic medical centers, with notable diversity in professional role, which allows findings to capture the perspectives of many multidisciplinary stakeholders and enhances transferability of findings. This study addresses a critical gap in the developing body of literature on psychosocial aspects of orthopedic care and recovery.

### Limitations

Our findings should be considered in light of important limitations. Our sample included predominantly white and male health care professionals, especially among surgeons and residents. While the demographic profile of our sample is representative of the population of orthopedic health care professionals in the U.S. [37], it is essential to note that our findings likely do not capture the range of perspectives of all orthopedic professionals. We also did not collect data in a way that allowed us to explore patterns in perceptions based on participant demographic characteristics. In addition, our sample only included health care professionals who work in orthopedic clinics. Future work is needed to capture the perspectives of stakeholders at the level of the organizations and systems (e.g., hospital leaders, policy makers). Because successful integration requires leadership support, their perceptions and recommendations would help promote a larger-scale endorsement of multidisciplinary care in orthopedic settings. Finally, our study did not capture the perspectives of patients in orthopedic settings. Many qualitative findings we report represent orthopedic providers’ perceptions of patient experiences and preferences (e.g., comfort discussing mental health with medical providers). It is essential to verify these perceptions and characterize patients’ perspectives on integrating psychosocial services into orthopedic care to inform the development of integrated care initiatives that are acceptable and accessible to patients.

## Conclusion

Integrated psychosocial care for orthopedic trauma patients has the potential to improve patient recovery, long-term physical and mental health outcomes, and satisfaction with care. However, multidisciplinary support is necessary for successful integration. We conducted focus groups with orthopedic health care professionals to characterize their perceptions and recommendations regarding integrated psychosocial services for orthopedic care. Findings can help inform the development and implementation of initiatives to integrate psychosocial services within orthopedic settings.
